# Congenital Nail Disorders among Children with Suspected Ectodermal Dysplasias

**DOI:** 10.3390/genes13112119

**Published:** 2022-11-15

**Authors:** Sigrun Maier-Wohlfart, Carmen Aicher, Ines Willershausen, Nicolai Peschel, Udo Meißner, Lina Gölz, Holm Schneider

**Affiliations:** 1 Center for Ectodermal Dysplasias Erlangen (CEDER), University Hospital Erlangen, 91054 Erlangen, Germany; 2 Department of Pediatrics, Friedrich-Alexander-Universität Erlangen-Nürnberg, 91054 Erlangen, Germany; 3 Department of Orthodontics and Orofacial Orthopedics, University Hospital Erlangen, 91054 Erlangen, Germany; 4 Independent Researcher, 96047 Bamberg, Germany

**Keywords:** congenital nail disorders, ectodermal dysplasia, anonychia/hyponychia congenita, R-spondin 4 (*RSPO4*)

## Abstract

We report on a cohort of 204 children referred between January 2017 and January 2022 to the German Center for Ectodermal Dysplasias, Erlangen. The most frequent reasons for referral were tooth malformations and lack of multiple teeth leading to the suspicion of an ectodermal dysplasia. Many patients also suffered from being unable to perspire. Nail abnormalities, in contrast, represented a much rarer finding, albeit the impact on some individuals was large. As ectodermal dysplasias are congenital genetic conditions affecting the development and/or homeostasis of two or more ectodermal derivatives, including hair, teeth, nails, and certain glands, we analyzed congenital nail disorders detected in these patients. Dystrophic or otherwise abnormal nails were evident in 17 of 18 subjects with pathogenic *WNT10A* or *GJB6* variants but in none of 161 children with *EDA* variants underlying X-linked hypohidrotic ectodermal dysplasia. However, 2 of 17 children who carry mutations in *EDAR* or *EDARADD*, two other genes involved in the ectodysplasin A signaling pathway, showed nail abnormalities, such as brittle or hypoplastic nails. *TP63* variants were regularly associated with nail disorders. In one girl, anonychia congenita caused by a compound heterozygous variant of the R-spondin-4 gene (*RSPO4*) was diagnosed. Thus, nail dysplasia is rarer among patients with ectodermal dysplasia than commonly thought.

## 1. Introduction

Ectodermal dysplasias are a subgroup of genodermatoses, the entirety of genetic skin diseases, with X-linked, autosomal recessive, or autosomal dominant modes of inheri-tance. The molecular causes of most of them have been identified, elucidating how distinct alterations of signaling pathways can affect the normal embryonic development of ectodermal derivatives like skin, teeth, hair, nails, and certain eccrine glands (e.g., sebaceous, sweat or mammary glands). Although nail abnormalities are considered to be a common feature of many genodermatoses, their prevalence among patients with ectodermal dysplasia (ED) is unknown, as data from large cohorts have been difficult to collect. Furthermore, nail alterations may also be acquired (e.g., related to infectious and inflammatory disorders or trauma) and are often poorly described in the available case series [1,2]. This is in contrast to the fact that, collectively, hereditary, and acquired nail disorders account for 3–11% of pediatric dermatology consultations [3,4]. Congenital nail abnormalities can occur isolated or as a component of genetic syndromes and may involve changes in nail color, texture, and/or shape [5]. In our experience, they do not only facilitate differential diagnosis, but their clinical relevance, particularly for females, is also underestimated.

This paper summarizes the nail-related findings in a large cohort of pediatric patients with clinically suspected ED. X-linked hypohidrotic ectodermal dysplasia (XLHED; Christ–Siemens–Touraine syndrome; MIM #305100), the most frequent form of ED, is characterized by a symptom triad of hypotrichosis, hypo- or even anodontia, and hypo- or anhidrosis, caused by pathogenic variants of the gene *EDA* (MIM *300451) which encodes the signaling protein ectodysplasin A1. Autosomal recessive and autosomal dominant forms of hypohidrotic ED are often evoked by pathogenic variants of either *EDAR* (MIM *604095) coding for the ectodysplasin A receptor (EDAR) or *EDARADD* (MIM *606603) encoding the EDAR-associated death domain adapter. The phenotype may be very similar to that of XLHED due to the common molecular signaling pathway [6,7,8]. Patients with Incontinentia pigmenti (IP; MIM #308300), a rare X-linked disorder caused by pathogenic variants of the gene *IKBKG* (MIM *300248) display a characteristic skin blistering during early infancy followed by hyperpigmentation, tooth anomalies, sparse hair, and sometimes severe involvement of the eyes and the central nervous system [9]. In hemizygous males, IP is associated with severe immunodeficiency which is usually lethal. Pathogenic *WNT10A* (MIM *606268) variants can be responsible for isolated tooth agenesis as well as for odonto-onycho-dermal dysplasia (OODD; MIM #257980) and Schöpf–Schulz–Passarge syndrome (SSPS; MIM #224750). Phenotypic features of recessively inherited OODD and SSPS include hypodontia, nail dystrophy, and palmoplantar hyperkeratosis [10,11,12]. Pathogenic variants of *GJB6* (MIM *604418) underlie Clouston syndrome (MIM #129500), an autosomal dominant disorder with the key symptoms nail dystrophy, alopecia, and palmoplantar hyperkeratosis [13]. 

Moreover, there are complex ED syndromes, such as ankyloblepharon-ectodermal dysplasia-cleft lip/palate (AEC) syndrome (MIM #106260) and ectrodactyly-ectodermal dysplasia-cleft lip/palate (EEC) syndrome (MIM #604292), two dominantly inherited disorders caused by pathogenic variants of the gene *TP63* (MIM *603273) that encodes a key transcription factor, tumor protein 63 (p63). The clinical findings in patients with p63-related syndromes include alopecia, dystrophic nails, hypodontia, and (often bilateral) cleft lip and/or palate. In addition, AEC syndrome is characterized by congenital anomalies of the eyelids, while ectrodactyly of hands and feet is a specific feature of EEC syndrome [14,15]. Relevant hypohidrosis does not belong to the regular symptoms associated with *TP63* mutations [16].

The absence or maldevelopment of finger- and toenails is the main characteristic of anonychia congenita and its milder phenotypic variant, nonsyndromic congenital nail disorder-4 (NDNC4; MIM #206800). Both are autosomal recessive disorders due to pathogenic variants of *RSPO4* (MIM *610573) encoding roof plate-specific (R)-spondin 4. They show a variable phenotype, ranging from the complete absence of the nail field to one of reduced size, with or without a rudimentary nail [17,18,19]. The R-spondin 4 protein is expressed in the nail mesenchyme and acts as an activator of the Wnt/β-catenin signaling pathway [20,21].

Continuous referral of patients with all forms of ED to our center and their systematic clinical examination allowed a retrospective compilation of nail disorders seen during the last five years. 

## 2. Subjects and Methods

### 2.1. Patients and Study Design

A cohort of 204 children (both sexes) referred between January 2017 and January 2022 with clinically suspected ectodermal dysplasia to the German Center for Ectodermal Dysplasias Erlangen (CEDER) was investigated. The inclusion criterion was the presence of abnormalities affecting at least two different ectodermal structures. Those patients who had not yet obtained a molecular genetic diagnosis were enrolled in the study “Detection and functional investigation of potentially pathogenic variants of HED-associated genes and new candidate regions”, approved by the Ethics Committee of the Friedrich-Alexander-Universität Erlangen-Nürnberg. Patients referred because of tooth malformations and lack of multiple teeth were usually seen in a joined pediatric and dental clinic. In all patients and in some of their family members if indicated, the nail phenotype (size, shape, color, fragility, thickness, signs of onycholysis) was assessed, focusing on congenital nail defects. Localized abnormalities that did not affect the majority of finger- or toenails were not considered. Nail disorders were documented during the first presentation at our center by photographs of both hands and feet. The study participants and/or their legal guardians provided written informed consent to the use of DNA for genetic analysis and further research. 

### 2.2. DNA Analysis

Standard gene variant analysis (DNA isolation from blood, polymerase chain reaction, subsequent Sanger sequencing, or multiplex ligation-dependent probe amplification) was performed as described previously [22]. Oligonucleotide primer sequences and thermal cycling conditions are available upon request. The software program ChromasPro 2.1.9 (Technelysium Pty Ltd., South Brisbane, Australia) was used for analysis of the electropherograms. In two cases, samples were sent to a provider of next-generation sequencing services (CeGaT GmbH, Tübingen, Germany) for whole-exome sequencing (WES) using the Illumina NovaSeq6000 Sequencing Systems. Datasets were analyzed bio-informatically with the Golden Helix GenomeBrowse tool (Golden Helix, Bozeman, MT, USA). Each detected variant was assessed with the mutation prediction tools Mutation Taster (Charité, Berlin, Germany; Cardiff University, Cardiff, UK) and/or the Ensembl Variant Effect Predictor also containing SIFT and PolyPhen-2 scores for protein changes (European Molecular Biology Laboratory’s European Bioinformatics Institute, Hinxton, UK). 

### 2.3. In Silico Protein Structure Analysis

For structure prediction of pathogenic variants of the RSPO4 protein (UniProt: Q2I0M5), three-dimensional model data from AlphaFold (https://α-fold.ebi.ac.uk/ accessed on 16 April 2022) were analyzed with AlphaFold Colab (https://colab.research.google.com/github/deepmind/alphafold/blob/main/notebooks/AlphaFold.ipynb accessed on 16 April 2022; [23]). UCSF ChimeraX was used for the visualization of the molecular graphics (resource for biocomputing, visualization, and informatics at the University of California [24]).

## 3. Results

**Figure 1:**
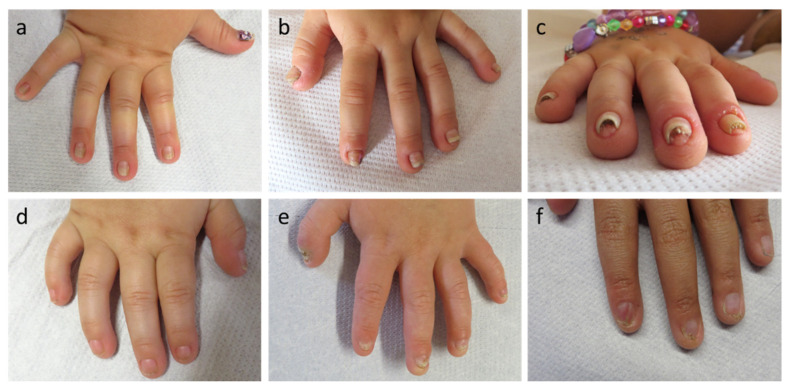
Exemplary nail abnormalities observed in the patient cohort. (**a**–**c**) Dystrophic nails of patients with Clouston syndrome; (**d**–**f**) Spectrum of nail plate abnormalities in p63-associated syndromes.

A total of 26 (12.8%) of 204 children with clinically suspected ED had nail abnormalities, such as brittle, hypoplastic or completely missing finger- and/or toenails, at the time of first presentation at our center (Table 1). The nails were regularly affected only in patients with Clouston syndrome (two females; Figure 1a–c), AEC syndrome (one male and three females; Figure 1d–f) and NDNC4 (one female). Approximately 30% of all patients did not yet have a molecular diagnosis of their condition and required molecular genetic analysis. Most individuals with pathogenic *WNT10A* variants underlying OODD or SSPS displayed characteristic nail dystrophies (six males and nine females; 94%), one infant with numerous missing teeth and moderate skin issues had normal nails. EEC syndrome was associated with nail abnormalities in two out of three male patients. However, not a single one of 161 male or female patients with XLHED and none of the three girls with IP showed obvious peculiarities of their finger- and toenails, while two female patients with autosomal recessive or dominant hypohidrotic ED caused by *EDAR* and *EDARADD* mutations, respectively, presented with dystrophic toenails (Table 1). 

In cases of Clouston syndrome, typical nail plate malformations included a narrow and dysmorphic shaping, hyperconvexity, milky white discoloration, and thickening of the nails with distal onycholysis (Figure 1a–c). Nail plates of the patients with AEC or EEC syndrome were partially discolored, fragile, frayed at the distal edges and showed ptery-gium formation and underdeveloped cuticles (Figure 1d–f). 

**Figure 2:**
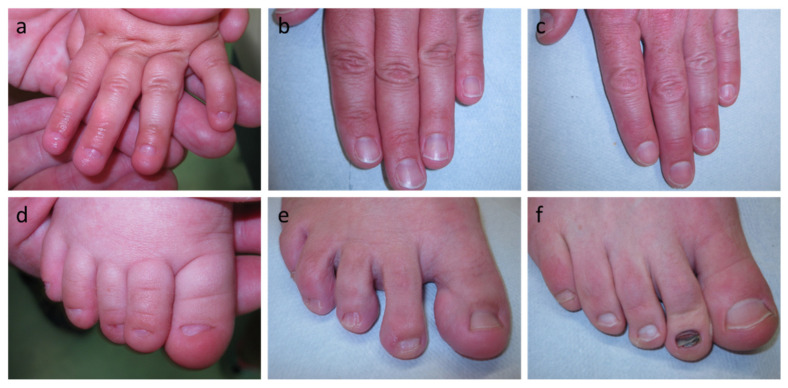
Phenotypic findings in the case of anonychia congenita (**a**–**c**) Fingernails of the index patient, her mother and her father; (**d**–**f**) Toenails of the index patient, her mother and her father.

One child later diagnosed with anonychia congenita, a two-year-old girl born to non-consanguineous and phenotypically unremarkable parents, was referred to the CEDER because of missing finger- and toenail development. Absence of all nail plates had been observed from birth, but did not relevantly affect grasping or other functions of the hands. Nail bed, matrix, and fold and the surrounding skin seemed to be unaffected and had a healthy appearance (Figure 2a,d). Except for hypoplastic nail plates on the big toes, the mother showed no nail abnormalities (Figure 2b,e). Four toes of the father’s right foot displayed somewhat brittle nails with a trauma-induced subungual hematoma on the second toe (after running a marathon; Figure 2f), whereas the other finger- and toenails were unremarkable (Figure 2c,f). The infant was able to sweat normally, but had rather sparse eyebrows. 

**Figure 3:**
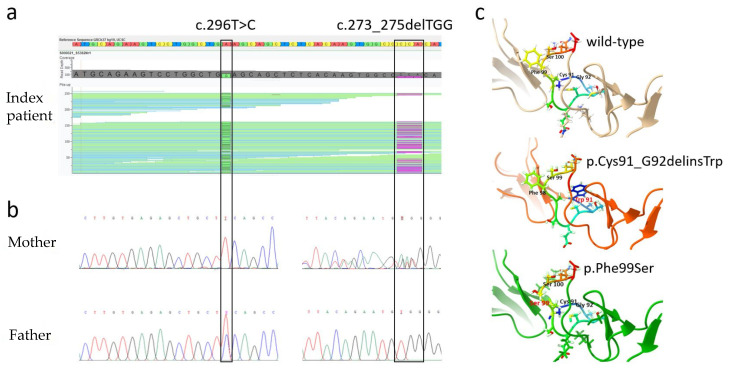
Molecular genetics of the (familial) case of anonychia congenita. (**a**) Golden Helix GenomeBrowse analysis of the WES results showing the two probably pathogenic *RSPO4* variants c.296T>C and c.273_275delTGG (reverse complement DNA sequence) of the compound-heterozygous index patient; (**b**) Chromatograms of the parents’ Sanger sequencing results displaying the heterozygous carrier status of mother and father for *RSPO4* c.273_275delTGG and c.296T>C, respectively; (**c**) Predicted three-dimensional structures of wild-type RSPO4, mutant p.Cys91_Gly92delinsTrp and p.Phe99Ser proteins.

Whole-exome sequencing of leukocyte-derived DNA from the index patient was performed. Analysis of the obtained bioinformatic data, particularly with regard to potential pathogenicity of detected variants and known associations of candidate genes with the patient’s phenotype, revealed few regions of interest. Two heterozygous and (probably coincidentally) adjacent alterations in the gene *RSPO4*, the variant c.296T>C (p.Phe99Ser) and the in-frame deletion c.273_275delTGG (p.Cys91_Gly92delins-Trp), turned out to be the most relevant changes in the exome of the affected girl (Figure 3a).

While the variant *RSPO4* c.296T>C is novel and predicted to be disease-causing, a report on *RSPO4* c.273_275delTGG has already been included in the Exome Aggregation Consortium (ExAC) database for heterozygous carriers. Variant effect prediction tools classify it as “likely polymorphic”. We assume that both variants add up to cause the infant’s phenotype in a compound-heterozygous manner. Sanger sequencing of genomic DNA of the parents revealed the mother to carry the deletion and the father to be a carrier of the missense variant, each in a heterozygous state (Figure 3b). The variant p.Cys91_Gly92delinsTrp is predicted to disrupt the normal disulfide bond formation bet-ween amino acids 91 and 98 of the wild-type protein (Figure 3c). The phenylalanine at position 99 is highly conserved and neighboring p.Ser100, an amino acid known to be important for the regulation of the Wnt/β-catenin signaling pathway due to its interaction with the leucine-rich repeat-containing G-protein-coupled receptors (LGR) 4, 5, and 6 [25].

In addition, the index patient was compound-heterozygous for two variants of the related gene *LGR5* (c.1997T>C/p.Val666Ala and c.2125C>G/p.Pro709Ala) which might also play a role in combining anonychia congenita with a mild hair phenotype.

## 4. Discussion

Nail disorders may have various phenotypic presentations, ranging from harmless discolorations to complete absence of all nails, as well as different causes, including genetic conditions, injuries, malnutrition or infections [26]. Depending on the severity, they impact the quality of life by causing psychological and/or functional problems [27]. Nail deformities and/or dystrophies are well known features of some ED entities, such as OODD/SSPS, Clouston syndrome, and AEC/EEC syndrome, which was confirmed by our findings. The nail phenotype may be rather specific like in patients with Clouston syndrome or show a wider spectrum of nail plate abnormalities as observed in children with *WNT10A* or *TP63* mutations. In cases of XLHED/HED, diverging information is available in the literature. Some authors stated that nail anomalies are generally not seen in subjects with XLHED, others report about 50% of their patients being affected [28,29,30]. This discrepancy could possibly be explained by subjective definitions of nail defects, especially in mild cases [31]. The complete absence of obvious nail abnormalities in our large cohort of pediatric patients with XLHED, however, indicates that this ED entity does not frequently involve the nails, at least in children. Nail defects become evident with age in some cases, but are usually not severe. Raising awareness and knowledge among patients and physicians is likely to improve the documentation of nail-related issues and will, therefore, lead to more standardized and comparable assessments in future studies. 

The prevalence of pure nail disorders is probably underestimated, as patients do not always seek medical advice and abnormalities can easily be missed by physicians. Rare conditions of skin appendages have received relatively little scientific attention so far and no therapies are available for the hereditary forms of isolated nail disorders [32]. Pathogenic variants of five genes (*RSPO4*, *FZD6*, *HPGD*, *PLCD1,* and *COL7A1*) have been reported to cause congenital pure nail disorders, among which *FZD6* (frizzled class receptor 6) and *RSPO4* are responsible for most of the cases [18,20,33,34]. Since hair is usually not affected by such mutations, the patient with anonychia congenita described in this paper was included in the study because of his sparse eyebrows in addition to the missing nails. RSPO4 functions as an agonist binding to the Frizzled receptor family which is involved in the activation of the Wnt/β-catenin signaling pathway. This well-known pathway plays a crucial role in the development of various ectodermal derivatives. LGR4–6 serve as receptors for RSPO proteins and have been assumed to connect with a Frizzled/low-density lipoprotein receptor-related protein co-receptor complex [34,35]. Mediation of the Wnt/β-catenin signaling pathway by RSPO4 seems to be LGR-dependent [25]. With this in mind, the two additional variants of the gene *LGR5* found in the patient with *RPSO4*-associated anonychia congenita are certainly very interesting, although a potential pathomechanism remains elusive.

Although the genetic causes of some isolated nail disorders have not yet been identified, the increasing usage of next-generation sequencing in molecular diagnostics is likely to enable the detection of pathogenic variants of other relevant genes within the next few years [32]. The same applies to complex genetic conditions, such as the ectodermal dysplasias.

## 5. Conclusions

Nail abnormalities seem to be accessory, sometimes also severe symptoms in the group of patients with pathogenic variants of the genes *WNT10A*, *GJB6*, *TP63*, and *RSPO4*, but appear to play a minor role in autosomal recessive or dominant forms of HED. In our large cohort of pediatric patients, *EDA* variants underlying XLHED were not associated with obvious congenital nail disorders. Physicians working in the field of pediatric dermatology as well as dentists and clinical geneticists should be sensitized to this difference, as nail-related findings—similar to disease-specific schemes of missing teeth—may facilitate or even guide the differential diagnosis. 

## Figures and Tables

**Table 1:** Proportion of nail abnormalities among children with clinically suspected ED seen at the CEDER in the last five years.

Disease(s)	Affected Gene(s)	Number of Patients Seen 2017–2022	Patients with Nail Abnormalities
Hypohidrotic ED, X-linked	*EDA*	161	0 (0%)
Hypohidrotic ED, autosomal dominant or recessive	*EDAR, EDARADD*	14	2 (14.3%)
Incontinentia pigmenti	*IKBKG*	3	0 (0%)
Odonto-onycho-dermal dysplasia/ Schöpf-Schulz-Passarge syndrome	*WNT10A*	16	15 (93.8%)
Clouston syndrome	*GJB6 2*	2	2 (100%)
Ankyloblepharon-ED-cleft lip/palate (AEC) syndrome	*TP63*	4	4 (100%)
Ectrodactyly-ED-cleft lip/palate (EEC) syndrome	*TP63*	3	2 (66.7%)
Anonychia congenita	*RSPO4*	1	1 (100%)

## Data Availability

Not applicable.
